# A Machine Learning-Based Method for Developing the Chinese Symptom Checklist-11 (CSCL-11)

**DOI:** 10.3390/bs15040459

**Published:** 2025-04-02

**Authors:** Xuanyi Cai, Yunan Zhang, Meng Su, Fan Chang, Lei Quan, Yixing Liu, Bei Wang

**Affiliations:** 1 School of Management, Beijing University of Chinese Medicine, Beijing 102488, China; 20220935337@bucm.edu.cn; 2 School of Life Sciences, Beijing University of Chinese Medicine, Beijing 102488, China; 20240941099@bucm.edu.cn (Y.Z.);; 3 University Student Mental Health Education and Counseling Center, Beijing University of Chinese Medicine, Beijing 102488, China

**Keywords:** Chinese version of SCL-90, variable clustering, machine learning, screening instrument

## Abstract

The Chinese version of the Symptom Checklist-90 (SCL-90) is excessively lengthy, resulting in extended completion time and reduced respondent compliance. This study aimed to utilize a condensed subset of items from the Chinese SCL-90 to identify individuals at high risk for psychological disorders based on machine learning methods, forming a concise and efficient preliminary psychopathological screening instrument for the Chinese general population. Analyzing data collected from 4808 SCL-90 psychological surveys, this study applied variable clustering to select the most representative items, resulting in an 11-item scale: the Chinese Symptom Checklist-11 (CSCL-11). The CSCL-11 demonstrated high internal consistency (Cronbach’s α = 0.84). The results of factor analysis supported a single-factor model for the CSCL-11, demonstrating an acceptable fit (SRMR = 0.035, RMSEA = 0.064, CFI = 0.935, and TLI = 0.919). The CSCL-11 demonstrated strong predictive performance for the Global Severity Index (GSI; RMSE = 0.11, R^2^ = 0.92, Pearson’s r = 0.96) and various subscale scores (RMSE < 0.25, R^2^ > 0.70, Pearson’s r > 0.85). Additionally, it achieved a 96% accuracy rate in identifying individuals at high risk for psychological disorders. The comparison results indicated that the CSCL-11 outperformed SCL-14, SCL-K11, and SCL-K-9 in predicting GSI scores. In identifying high-risk groups, CSCL-11 demonstrated performance similar to that of SCL-14 and surpassed both SCL-K11 and SCL-K-9. The CSCL-11 retains most of the critical information from the original Chinese SCL-90 and serves as a preliminary psychopathological screening tool for the Chinese general population.

## 1. Introduction

As one of the most widely used self-report mental health scales, the Symptom Checklist 90 (SCL-90) has been translated into various languages and is widely used in many countries to assess psychological and physiological symptoms within a week (Müller et al., 2009). The SCL-90 was initially created by Derogatis et al. (1973), and later revisions were made to two items in the anxiety subscale, creating the Symptom Checklist-Revised-90 (SCL-90-R) (Derogatis, 1992). The SCL-90-R has demonstrated good internal consistency, with Cronbach’s alpha coefficient > 0.7 for the total scale and ranging from 0.76 to 0.9 for subscale scores (Ransom et al., 2010). Also, previous research indicated high correlations between the SCL-90-R and other scales, such as the Beck Depression Inventory (BDI) and Minnesota Multiphasic Personality Inventory (MMPI), suggesting its good convergent validity (Brophy et al., 1988; Derogatis et al., 1976).

However, the SCL-90 and SCL-90-R were shown to have excessive length and redundancy in items (Shang, 2023; Prinz et al., 2013), typically requiring 12–15 min for completion (Derogatis et al., 1973; Z. Yu et al., 2024). The survey’s length may reduce compliance and response accuracy due to respondent fatigue and decreased focus (Lundqvist & Schröder, 2021). To address these limitations, the Brief Symptom Inventory (BSI) was proposed in 1975 as the first shortened version of the SCL-90-R (Derogatis, 1993). Over the subsequent decades, researchers from different countries have developed various short versions of the SCL-90-R tailored for different contexts. The majority of these simplified scales were obtained with factor analysis, retaining items with higher loadings on the corresponding factor, such as BSI (Derogatis, 1993), BSI-18 (Derogatis, 2000), SCL-27 (Hardt & Gerbershagen, 2001), SCL-10N (Nguyen et al., 1983), SCL-10R (Rosen et al., 2000), SCL-6 (Rosen et al., 2000), and SCL-3/7 (Timman & Arrindell, 2022). Additionally, based on the BSI, Lutz et al. (2006) selected items that exhibited strong correlations with validation tools such as the General Severity Index (GSI), Beck Depression Inventory (BDI), and the subscales of SCL-90-R (i.e., somatization, depression, anxiety, and phobic anxiety). Subsequently, the selected items demonstrating significant test-retest reliability and sensitivity to temporal change were further refined to create the SCL-K11. While these methods improve efficiency and demonstrate validated effectiveness, most of the simplified versions do not comprehensively cover all dimensions of the SCL-90. For instance, SCL-K11 primarily includes items from the depression and anxiety dimensions, potentially limiting its ability to fully represent all the information conveyed by the SCL-90-R. Different from previously mentioned short versions with items selected from the whole scale, the SCL-K-9 (Klaghofer & Brähler, 2001) was developed by selecting items from each subscale of the original SCL-90-R that demonstrated the highest correlation with the Global Severity Index (GSI). Therefore, unlike other versions that tend to emphasize specific dimensions, the SCL-K-9 maintains a more balanced representation of information from each subscale of the SCL-90-R. However, the SCL-K-9 extracts only one item from each dimension, which may not fully capture the complex information within each dimension and may overlook the interrelationships between dimensions, given the high correlations among the dimensions of the SCL-90-R. It is worth noting that most of these brief versions were developed based on data from specific populations during the late 20th and early 21st centuries. With the passage of time, individuals’ mental health may undergo changes. Therefore, it is important to consider simplifying the Chinese version of SCL-90 using novel methods based on the mental health status of contemporary populations, enhancing the relevance and representativeness of the simplified version in today’s society (Z. Yu et al., 2024; Xu et al., 2024).

While the prior abbreviated versions maintain the accuracy and screening efficiency of the SCL-90-R, their representation of the original psychological dimensions is lacking and scope is inadequately represented. These versions tend to overemphasize certain dimensions, such as anxiety and depression, while inadequately addressing others, such as somatization and phobic anxiety. Furthermore, the selection of items may incorporate elements that are very simple, which reduces the overall predictive power and reliability of the assessment. Furthermore, the previous attempts at simplification were too radical and relied on a completely exclusionary approach of entire missing dimensions or using poorly chosen items, which left unfilled gaps needed to assess the contemporary population’s mental health. This is the focus of this study: developing a more comprehensive item selection aimed at enhancing the validity of the assessment while also utilizing an advanced approach that employs robust underlying principles grounded in data.

The development of machine learning techniques offers advantages in efficiently handling massive, high-dimensional data, which are gradually applied to research on scale simplification (Shang, 2023; Liu, 2021). In studies investigating the abbreviation of the Chinese version of the SCL-90, Liu (2021) devised a dynamic simplification scheme using the Gradient Boosting Regression Tree (GBRT) algorithm, resulting in an average reduction of 23 items. Concurrently, Shang (2023) employed the FP-Growth algorithm and the Stacking_LSGL ensemble learning model to reduce the original 90 items to 26. These two supervised learning algorithms ensured satisfactory predictive accuracy for subscale scores, with sensitivity and specificity exceeding 80%. In 2024, Z. Yu et al. (2024) utilized the support vector classifier (SVC) algorithm to simplify the SCL-90 from 90 items to 29 while maintaining specificity and sensitivity above 85%. These studies have taken significant initial steps in simplifying the Chinese version of SCL-90. However, their simplification strategies relied on assessing inter-dimensional predictive accuracy to eliminate entire dimensions that were easier to predict, followed by item reduction within the remaining dimensions. Although this approach preserves relative accuracy, it disregards substantial information conveyed by the excluded dimensions.

Despite significant progress in prior research on scale simplification, challenges related to the structural validity of the SCL-90-R remain, with multiple studies indicating difficulty in replicating its nine-factor structure (Hessel et al., 2001). Therefore, instead of selecting items from each subscale, we utilized an unsupervised machine learning algorithm known as variable clustering to choose the most representative items from the entire SCL-90 scale. Variable clustering utilizes correlation coefficients to construct a correlation matrix, identifying highly correlated items within the dataset. It then applies cluster analysis to divide these items into groups with similar features. Following clustering, we selected the most representative items from each cluster to form a new shortened version. This process effectively addresses multicollinearity issues arising from strong inter-item correlations by reducing redundant information and ensuring that each representative item within a cluster maximally retains the essential information of the original variables. Variable clustering is particularly suitable for high-dimensional and highly correlated data structures, making it a suitable approach for the context of the SCL-90, where items exhibit strong correlations.

This study aims to enhance the construction of the Chinese version of SCL-90 by unsupervised machine learning. It uses variable clustering to determine the most representative items in the scale that summarizes the SCL-90 best. The primary goal is to accurately retain the information conveyed by the original instrument’s multidimensional structure while adapting it for current users alongside ensuring validity and utility. Previous methodologies mainly depended on factor analysis or supervised learning models where dimensions are deleted after selection. This novel approach, which applies clustering to highly correlated data, helps remove redundant information while simultaneously ensuring that the final shortened scale is both comprehensive and efficient. This method, in relation to previous approaches, is less stringent and more accurate, preserving critical components from SCL-90 while optimizing other aspects of the psychological assessment.

To ensure the applicability of this new shortened version of the SCL-90, we first evaluated its factor structure and internal consistency. Following this, we evaluated its ability to predict the GSI and subscale scores of the original SCL-90. Furthermore, we aimed to develop a superior machine learning classifier that can identify individuals at high risk of psychological disorders with the selected subset of items. Building upon models used in previous SCL-90-R simplification studies and recent machine learning prediction research (Z. Yu et al., 2024; Liu, 2021; Chiew et al., 2019; Kopitar et al., 2020; Kosmicki et al., 2015), we selected five high-performing machine learning models (i.e., logistic regression, random forest, adaptive boosting, gradient boosting decision tree, and support vector classifier) for comparative analysis. By comparing the five machine learning predictive models, we selected the optimal classifier along with the new brief version for rapid psychopathological screening in future applications. Finally, to assess the quality and practicality of this new version of SCL-90, we compared its predictive accuracy with that of previously proposed abbreviated versions of the SCL-90-R in forecasting both the scale (subscale) scores and identification of high-risk populations for psychological disorders.

Machine learning is one of the core tools for data analysis in the field of artificial intelligence (AI). Its fundamental principle is to leverage the powerful computational capabilities of computers to learn from large datasets, continuously training and iterating to produce an effective learner. In this manuscript, we delineate the use of machine learning, particularly unsupervised machine learning (like variable clustering), to optimize the selection of the most informative items on the SCL-90 scale, with the goal of increasing the efficiency and accuracy of the resultant mental health screening tool.

1. The newly shortened scale effectively encapsulates the dimensional information of the full instrument, thereby providing a unidimensional yet efficient and representative tool for psychological assessment.
2. The variable clustering method, which identifies the most representative items, was utilized for the study. This method is within the category of unsupervised machine learning algorithms. It resolves multicollinearity and redundancy issues in datasets by selecting items that hold the most information, thus making the items less informative.
3. Unlike the prior abbreviated versions that focused on specific dimensions, the newly shortened scale aimed to incorporate all the major psychological aspects portrayed in the SCL-90.
4. Item selection using variable clustering as an unsupervised machine learning technique is an innovative method compared to the rest, which leaned more towards supervised learning algorithms or factor analysis to create their items. These methods are devoid of accuracy and valuable data and are rigid at the same time.

There is a guarantee of item selection that breaks free from the traditional dimensional constraints of the SCL-90 due to the novel way adopted by the study, which allows for a more comprehensive psychological screening tool.

## 2. Materials and Methods

### 2.1. Sample

All currently enrolled students from a large public university in North China were invited to participate in a mental health survey via an online platform conducted by the university’s psychological center. Consequently, a total of 4827 undergraduate and graduate students completed the original Chinese version of SCL-90. Questionnaires with missing values, as well as those where all items received the same response, were excluded during the data preprocessing stage, resulting in a final dataset of 4808 complete and valid responses. Ethical approval for this study was obtained from the Medical Ethics Committee of Beijing University of Chinese Medicine. All students participated in the psychological survey voluntarily and provided their informed consent online. The mean age of the students was 22.03 years (SD = 2.67). Out of the total, 75.7% of the students were female, and 23.4% were enrolled in the graduate stage.

### 2.2. Measures

The Chinese version of the SCL-90 was initially translated for use with clinical psychiatric patients (Wang, 1984) and later became widely used among the general public due to its sensitivity and practicality (Xie & Dai, 2006). Furthermore, the SCL-90 has been extensively employed in mental health surveys of college students, with normative data established for this population (Zhong & Li, 2009; Shi et al., 2013; Zhao et al., 2021). Additional studies have also explored the psychological and physiological dimensions of the SCL-90, reinforcing its comprehensive applicability in both clinical and general settings (Zhang et al., 2024; Chen et al., 2024). The SCL-90 includes dimensions such as Somatization (SOM), Obsessive-Compulsive (OC), Interpersonal Sensitivity (IS), Depression (DEP), Anxiety (ANX), Hostility (HOS), Phobic Anxiety (PHOB), Paranoid Ideation (PAR), Psychoticism (PSY), and Additional items (ADD), which assess appetite and sleep disorders (Tian et al., 2020). Each item of the SCL-90 was measured on a 5-point Likert scale (1 = “no problem” to 5 = “very serious”) (Wang, 1984). According to the findings of Y. Yu et al. (2020), the overall and subscale Cronbach’s alpha values for the Chinese version of SCL-90 ranged between 0.80 and 0.98, showing high internal consistency. Additionally, the following commonly utilized shortened versions were employed for comparative purposes: the SCL-27 (Hardt & Gerbershagen, 2001), BSI (Derogatis, 1993), SCL-14 (Prinz et al., 2013; Harfst et al., 2002), SCL-K11 (Lutz et al., 2006), and SCL-K-9 (Klaghofer & Brähler, 2001).

The Global Severity Index (GSI) is considered the best overall indicator for measuring overall psychological distress in the SCL-90, calculated as the average of item scores and used as the general index in this study (Tian et al., 2020; Y. Yu et al., 2020; Derogatis & Melisaratos, 1983). Converting the original GSI scores to T-scores (mean = 50, SD = 10), participants with GSI T-score ≥ 63 were considered the high-risk group for having clinically significant psychological distress (Tian et al., 2020). The GSI T-score threshold has been widely applied in recent psychological research and proven to be an effective and reliable indicator for identifying psychological disorders that require attention (Tian et al., 2020; Sereda & Dembitskyi, 2016; Monteiro et al., 2017; Shacham et al., 2009).

### 2.3. Method Background and Statistical Analyses

To maintain methodological standards, the statistical analyses were conducted with the aid of variable clustering integrated into supervised machine learning, along with exploratory and confirmatory factor analyses as well as model evaluation steps. Each part was focused on maximizing predictive accuracy as well as the generalizability of the newly developed abbreviated scale.

#### 2.3.1. Dataset Split

The entire dataset was set aside to cover eighty percent of the proportion for variable clustering, which was meant for scale reduction while preserving the dimensional information of the original constructs. The remaining 20% was reserved as test data for assessing the generalizability of the new scale and predictive models.

#### 2.3.2. Variable Clustering Analysis

Variable clustering analysis is a method used to reduce dimensionality by identifying and combining groups of closely interrelated variables. The goal is to detect latent constructs measured by a greater number of items while minimizing the scale without losing predictive power. The process begins by computing the correlation matrix for all items in the SCL-90 bounding box, followed by eigenvalue decomposition to detect latent structures. Based on the eigenvalue information, clusters are formed by regrouping items when the second maximum eigenvalue in a cluster surpasses a defined threshold. This threshold is iteratively adjusted in predefined step sizes to optimize the number of clusters. The key concepts involved in variable clustering are outlined below.

Correlation matrix (C): The correlation matrix, also referred to as C, is established to determine the extent to which the items are related. The matrix entry C_ij_ denotes the correlation between items i and j.

(1) $$C_{ij}=\frac{Cov\left(X_{i},X_{j}\right)}{\sqrt{Var(X_{i})\cdot Var(X_{j})}}$$

where Cov(X_i_, X_j_) is the covariance of X_i_ and X_j_, and Var(X_i_) and Var(X_j_) refer to the variance of X_i_ and X_j_, respectively.

Eigenvalue decomposition: To calculate the amount of variance each principal component accounts for, the eigenvalue decomposition of the correlation matrix is performed so that the eigenvectors (principal components) and eigenvalues may be obtained. Eigenvalues reveal the extent of variance contributed by each principal component. Clusters are created by aggregating items that share similar eigenvalue characteristics.

(2) Cv=λv

where C is the correlation matrix, v is an eigenvector, and λ is the eigenvalue of v.

RS_Ratio Metric (Sun et al., 2024): To determine the most representative item of each cluster, RS_Ratio metric is used. This metric assesses the ratio of the explained variance an item has within its cluster in comparison to the maximum explained variance of this item across all other clusters. The RS_Ratio is mathematically defined as follows:

(3) $${RS\_Ratio}_{ij}=\frac{1-\gamma_{ij}}{1-\nu_{ijmax}}$$

where γ_ij_ represents the proportion of variance for item j explained by the first principal component of cluster i, while ν_ijmax_ represents the maximum proportion of variance for item j, explained by the first principal components of all clusters except i. An item within a cluster that has the minimum RS_Ratio value is considered to have the strongest association with that cluster and the weakest association with other clusters. Therefore, we selected the item with the smallest RS_Ratio value within a cluster as its representative item.

#### 2.3.3. Supervised Machine Learning (SML) for Scale Development

Stand-alone, variable clustering does not indicate the optimal number of clusters. In a bid to ensure the simplified scale achieves a balance between parsimony and predictive power, variable clustering was combined with supervised machine learning models. The construction of the new scale was led by items expected to predict the Global Severity Index (GSI) scores. As a target for the simplified scale, it was required to achieve a Pearson correlation coefficient r ≥ 0.96 between the predicted and actual GSI scores in the test set, considering that among existing abbreviated versions of the SCL-90, the SCL-27 exhibits the highest Pearson correlation with the GSI (Pearson’s r = 0.96). (Hardt & Gerbershagen, 2001). Because GSI scores are integrated as the average of all items, regression models were constructed that included

- Ordinary Least Squares (OLS) Regression (Linear Regression)
- Robust linear regression
- Ridge regression (for regularization)

The simplest type of linear regression can be stated as

(4) $$Y=\beta_{0}+\beta_{1}X_{1}+\beta_{2}X_{2}+\cdots +\beta_{p}X_{p}+\epsilon$$

In this equation, Y is the dependent variable, which ranges from GSI score X_1_, X_2_, … X_p_ are the independent variables comprising the selected items, and β_0_ is the intercept, while β_1_, … β_p_ is the coefficients together with ϵ representing the error term.

The most accurate model was chosen in relation to measures and the number of items in the reduced scale was set after the given precision requirements were achieved. The measurement of model performance accuracy was confirmed by:

Root Mean Square Error (RMSE) (Z. Yu et al., 2024; Liu, 2021): The accuracy of the model is assessed using RMSE by measuring the difference between the predicted values ${\hat{Y}}_{i}$ to the real values Y_i_. The formula is as follows

(5) $$RMSE=\sqrt{\frac{1}{n}\sum_{i=1}^{n}{\left(Y_{i}-{\hat{Y}}_{i}\right)}^{2}}$$

where n is the number of observations.

Coefficient of Determination (R^2^) (Kopitar et al., 2020): R^2^, which is the Coefficient of Determination measures how much percentage of the variation in Y can be attributed to X. The formula is as follows:

(6) $$R^{2}=1-\frac{\sum_{i=1}^{n}{\left(Y_{i}-{\hat{Y}}_{i}\right)}^{2}}{\sum_{i=1}^{n}{\left(Y_{i}-{\bar{Y}}_{i}\right)}^{2}}$$

where ${\bar{Y}}_{i}$ is the average of the actual Y_i_ values.

Pearson’s Correlation Coefficient (r) (Hardt & Gerbershagen, 2001): r is commonly used to measure the linear relationship between predicted values and actual values. The formula is as follows:

(7) $$r=\frac{\sum_{i=1}^{n}\left(X_{i}-\bar{X})(Y_{i}-\bar{Y}\right)}{\sqrt{\sum_{i=1}^{n}{\left(X_{i}-\bar{X}\right)}^{2}\sum_{i=1}^{n}{\left(Y_{i}-\bar{Y}\right)}^{2}}}$$

#### 2.3.4. Factor Structure Validation (EFA and CFA)

Exploratory Factor Analysis (EFA): EFA is the method to explore the impacts of major factors behind an event without any kind of determined hypothesis about it. It is designed to identify the latent factors for a given set of variables. The factor model in EFA can be expressed as follows: X=ΛF+ϵ, where X is the matrix of observed variables, Λ is the factor loading matrix, F is the vector of factors, and ϵ is the vector of residuals.

For the purpose of exploring the factor structure of the scale created, an Exploratory Factor Analysis (EFA) was conducted on it based on half of the randomly extracted sample. The K-factors were set based on

- Kaiser–Guttman Rule (eigenvalues > 1)
- Cattell’s Scree Plot Test (Lebowitz et al., 2019; Pichardo et al., 2023; Spurgeon et al., 2015)

Confirmatory Factor Analysis (CFA): CFA is performed with the aim of confirming a predefined structure factor obtained after performing EFA. It aims to test if the data supports the proposed model. The structure of the CFA model is what was obtained for the EFA model, except that it has already defined factor structure. X=ΛF+ϵ, where the structure of Λ is constrained in terms of some theory or EFA results.

Considering the factor structure obtained from EFA, the other half of the sample was analyzed through Confirmatory Factor Analysis (CFA). The model fit was evaluated using (Hu & Bentler, 1999; Petrowski et al., 2019)

- Standardized Root Mean Squared Residual (SRMR) (<0.08)
- Root Mean Squared Error of Approximation (RMSEA) (<0.05 excellent fit, <0.08 acceptable fit)
- Comparative Fit Index (CFI) (>0.95 good fit, >0.90 acceptable fit)
- Tucker–Lewis Index (TLI) (>0.95 good fit, >0.90 acceptable fit)
- Chi-square test of model fit. However, it is sensitive to larger sample sizes (greater than 100) and often leads to the rejection of the null hypothesis, even when the model fit is acceptable (Lebowitz et al., 2019). Therefore, researchers place more emphasis on the fit indices mentioned above.

#### 2.3.5. Reliability Analysis

Reliability of psychological measurement examines the consistency and stability of scores produced by a given scale. In this research assessment, the reliability of the newly crafted abbreviated SCL-90 scale was conducted using Cronbach’s alpha and McDonald’s omega, which are metrics for internal consistency.

Cronbach’s alpha is the most popular measure for scale reliability, with 1 denoting perfect consistency and values below 0.7 indicating unreliable scales (Peterson, 1994). The formula for Cronbach’s alpha is presented as follows:

(8) $$\propto =\frac{N.\hat{c}}{\hat{v+\left(N-1\right)}.\hat{c}}$$

McDonald’s omega is considered a more robust measure of scale reliability than Cronbach’s alpha, especially when the assumption of tau-equivalence among items is violated. McDonald’s omega ranges from 0 to 1, with higher values indicating better internal consistency and values below 0.7 generally suggesting insufficient reliability. McDonald’s omega accounts for the varying contributions of individual items to the overall variance, thereby providing a more precise assessment of scale reliability. For a single-factor model, McDonald’s omega is computed as follows:

(9) $$\omega =\frac{{\left(\sum_{i=1}^{k}\lambda_{i}\right)}^{2}}{{\left(\sum_{i=1}^{k}\lambda_{i}\right)}^{2}+\sum_{i=1}^{k}\theta_{i}}$$

where $\lambda_{i}$ represents the factor loadings for each item, and $\theta_{i}$ denotes the corresponding error variances.

#### 2.3.6. Machine Learning for Classification

To utilize the new scale, we trained five machine learning models (Table 1) that attempted to classify individuals at risk of psychological disorders into high-risk categories.

For evaluating the models, ten-fold cross-validation with a five-fold grid search for hyperparameter tuning was used (Chiew et al., 2019). The models were assessed using accuracy, sensitivity (recall), precision, specificity, F1 score, and Matthews correlation coefficient (MCC). The mean and 95% confidence intervals (CI) of the evaluation metrics were calculated across the folds to identify the model with the best overall performance (Kopitar et al., 2020). Additionally, we calculated the area under the curve (AUROC, AUPRC) and plotted the Receiver Operating Characteristic (ROC) and precision–recall curves (PRC). Brier scores and calibration curves were used to evaluate how well predicted probabilities are aligned with actual outcomes. A lower Brier score indicates better model calibration, while a higher score suggests poor calibration or miscalibrated predictions. In consideration of the distribution of GSI scores, which are positively skewed and focus on high-risk identification, and given the focus on the identification of high-risk individuals, F1 and AUPRC were emphasized as metrics (Chiew et al., 2019).

#### 2.3.7. Evaluation Against Existing Versions

In order to assess the usefulness of the new scale, its predictive outcomes were evaluated against the widely used and representative shortened versions of the SCL-90-R, which are SCL-27, BSI-18, SCL-14, SCL-K11, and SCL-K-9.

Each version was implemented with the same regression technique and evaluated on the test dataset for RMSE, R^2^, and Pearson’s r. Furthermore, the classification performance was evaluated through accuracy, F1 score, and MCC. It is important to mention that previous versions classified the at-risk population with the use of GSI T-score ≥ 63 (Sereda & Dembitskyi, 2016).

The analyses were performed through Python 3.10.7 for the clustering analyses with ‘varclushi’ and sci-kit-learn for the machine learning model development.

## 3. Results

### 3.1. The Development of CSCL-11 and Its Prediction Performance for GSI and Subscale Scores

#### 3.1.1. The Development of CSCL-11 and Its Prediction Performance for GSI

**Figure 1:**
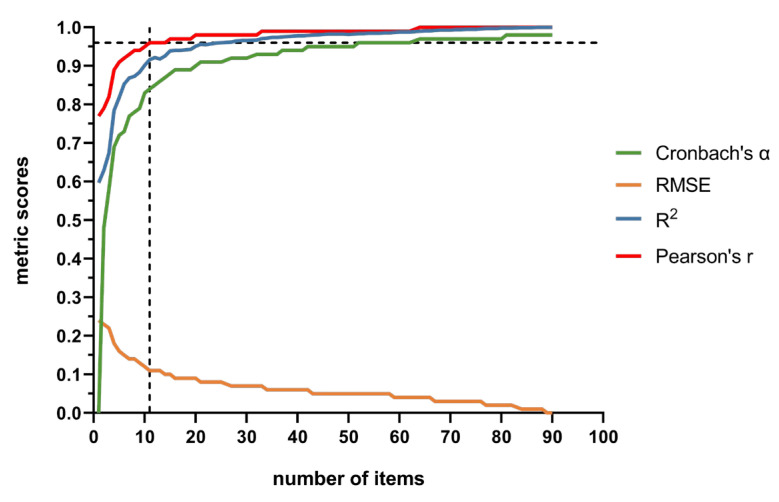
Internal consistency and predictive performance metrics during iterative process. (The dashed line indicates the number of items included in the abbreviated scale at the point where Pearson’s correlation coefficient reaches the threshold of 0.96).

During the combination of variable clustering with supervised machine learning, linear regression was selected as the preferred method because its predictive capacities and computational efficiency outperformed the robust linear regression and ridge regression. Figure 1 illustrates the changes in various metrics throughout the iterative process of variable clustering. As more items were incorporated into the scale, Cronbach’s alpha coefficient, R^2^, and Pearson’s r coefficient increased and then plateaued, while the RMSE demonstrated a stepwise decrease followed by stabilization. While incorporating additional elements into the model increases its internal consistency (i.e., Cronbach’s alpha) and predictive accuracy (i.e., RMSE, R^2^, Pearson’s r), the benefits stagnate after a certain threshold as well. This serves as proof of the optimum number of items on the simplified scale, wherein adding more items beyond this point does little to improve performance. This trend has considerable value when determining how many items should be included in the new shortened scale. When the current abbreviated scale included 11 items, the prediction error for the GSI in the test set was 0.11, with an R^2^ of 0.92 and a Pearson’s r of 0.96, meeting the setting threshold. Therefore, this study’s newly finalized abbreviated version (CSCL-11) incorporates 11 items from the original SCL-90, as detailed in Table 2. The CSCL-11 includes specific items from the subscales of somatization, obsessive-compulsive, depression, anxiety, hostility, phobic anxiety, paranoid ideation, psychoticism, and additional items. Notably, it does not include the items of interpersonal sensitivity, possibly because these items did not demonstrate sufficiently high correlations with other items within their own cluster or exhibited stronger correlations with items from other clusters.

#### 3.1.2. Factor Structure Validation and Reliability Analysis of the CSCL-11

**Figure 2:**
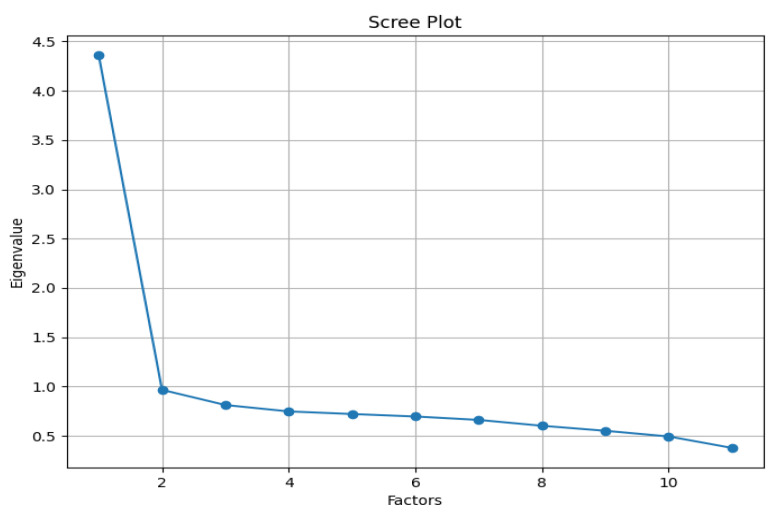
The Scree Plot of eigenvalues.

The Scree Plot shown in Figure 2 aids in determining the number of factors to retain by plotting eigenvalues against the number of factors. As illustrated, the eigenvalue of the first factor is markedly higher than those of subsequent factors, and it drops sharply before stabilizing at much lower levels, indicating that it accounts for the majority of the variance—a hallmark of unidimensionality. Furthermore, the distinct “bend” in the plot after the first factor further supports the notion that the scale is likely unidimensional. The single factor having an eigenvalue greater than one, as per the Kaiser–Guttman criterion, indicates with certainty that the data is made up of one factor, confirming once again that the scale is unidimensional. All standardized factor loadings ranged from 0.425 to 0.748 (see Table 3). Subsequent CFA fit indices indicated a good fit for the single-factor model, with SRMR = 0.035, RMSEA = 0.064, CFI = 0.935, and TLI = 0.919. Although the chi-square value was significant (*p* < 0.001), this was likely due to the large sample size, which increased the test’s sensitivity to minor discrepancies between the model and the data. Furthermore, the CSCL-11 demonstrated good internal consistency, with a McDonald’s omega value of 0.839 and a Cronbach’s alpha coefficient of 0.84, exceeding the satisfactory threshold of 0.7.

#### 3.1.3. Prediction Performance of the CSCL-11 in Predicting Subscale Scores

Table 4 indicates that the CSCL-11 demonstrated good predictive performance when using the linear regression method to predict the original SCL-90 subscale scores in the test set. According to the guideline, R^2^ values of 0.75, 0.50, and 0.25 correspond to robust, moderate, and weak levels of predictive accuracy (Kopitar et al., 2020). The CSCL-11 achieved robust predictive performance for most dimensions, except for the somatization dimension, which demonstrated a moderate level of predictive accuracy with an R^2^ value of 0.71. The RMSEs of the CSCL-11 in predicting these subscale scores were all less than 0.3, and all Pearson’s correlations between predicted scores and real scores were above 0.85.

### 3.2. The Predictive Performance of CSCL-11 for Identifying the High-Risk Group

**Figure 3:**
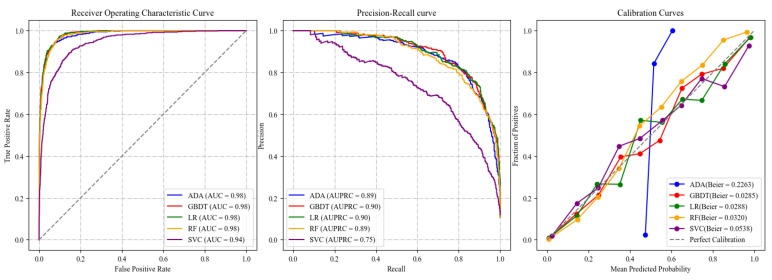
Receiver operating curves, precision–recall curves, and calibration curves of five candidate models.

**Figure A1:**
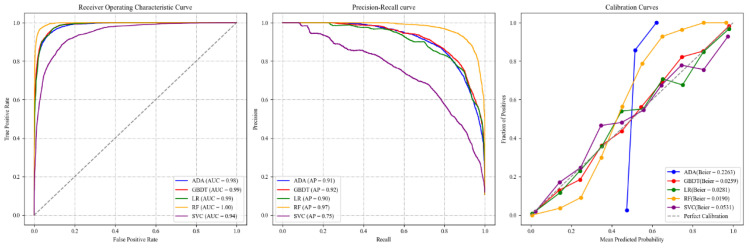
Receiver operating curves, precision–recall curves, and calibration curves of five candidate models in the training test.

Figure 3 displays the ROCs, PRCs, and calibration curves, respectively, for the five candidate models (i.e., logistic regression, random forest, adaptive boosting, gradient boosting decision tree, support vector classifier) for predicting the high-risk group of psychological disorders in the test set. The models’ performance on the training set is shown in Figure A1. All models achieved an AUROC above 0.94 and an AUPRC above 0.75. Among them, gradient boosting decision tree (GBDT) and adaptive boosting (ADA) exhibited the best overall performance, with both achieving an AUROC of 0.98 and an AUPRC of 0.90. Moreover, GBDT attained a Brier score of 0.0285, indicating that its predicted probabilities closely match the actual outcomes and thus reflect excellent probability calibration. A lower Brier score implies that GBDT is highly accurate in providing probabilistic estimates and predictions for high-risk individuals.

The calibration curves in Figure 3 compare the predicted mean probabilities from each model (on the *x*-axis) with the observed probabilities (on the *y*-axis). A well-calibrated model performs its predicted probabilities near the actual probabilities, which is depicted by the perfect calibration line (diagonal line). Among the models, the GBDT model appears well-calibrated as its calibration curve is close to the perfect calibration line, especially in the upper probabilities range. This demonstrates that the predicted probabilities from the GBDT model are highly reliable and closely match real probabilities. Logistic regression (LR) also shows relatively good calibration, although slightly imperfect than GBDT. The LR curve is near the ideal line, but small deviations indicate that predicted probabilities in certain areas may not be entirely trustworthy. Random forest (RF) predicted probabilities do not correspond well with outcomes in the mid to high interval range, as indicated by the notably less accurate calibration curve. Overestimation and underestimation predictions for specific outcomes suggest that the support vector classifier (SVC) model is less reliable due to the extreme deviation from the perfect calibration line at higher predicted probabilities. Meanwhile, ADA—despite its high AUROC and AUPRC—deviates considerably from the diagonal across much of the probability spectrum, suggesting that its probability estimates are less well-calibrated overall. Overall, GBDT, followed by logistic regression, exhibits the best calibration results on the graph, whereas RF, SVC, and adaptive boosting exhibit greater calibration difficulties, which negatively affects their reliability when making predictions at high probability intervals.

Table 5 summarizes their accuracies, sensitivities, specificities, precisions, F1 scores, and Matthews correlation coefficients (MCC). The gradient boosting decision tree (GBDT) was considered the best model overall in the study due to its balanced performance across multiple metrics, particularly in the context of identifying individuals at high risk for psychological disorders. While logistic regression showed slightly better precision and AdaBoost demonstrated a narrower confidence interval for the MCC, GBDT outperformed the other models in terms of overall consistency and predictive accuracy.

GBDT is a powerful ensemble learning method that builds a series of weak learners (decision trees) and improves performance iteratively by correcting errors made by previous models. This iterative correction process allows GBDT to handle complex, nonlinear relationships in the data, which is crucial when predicting psychological disorders, where relationships between symptoms are not always linear. Furthermore, GBDT achieved strong performance across key metrics such as accuracy, sensitivity (recall), and F1 score, all of which are important for identifying high-risk individuals in imbalanced datasets like this one. The model’s ability to balance both false positives and false negatives effectively made it especially valuable in screening for mental health conditions.

### 3.3. Comparison of CSCL-11 with Other Shortened Versions of SCL-90-R

Table 6 displays the dimensions corresponding to the items included in all abbreviated versions utilized in this study. Each version incorporates items from some of the dimensions from the original SCL-90, yet none cover all the subscales. As shown in Table 6, the depression subscale is involved in all versions, whereas the anxiety subscale is omitted only in the SCL-14. Except for the SCL-K11, all other abbreviated versions incorporate items from the somatization subscale. Moreover, unlike many other versions that exclude psychoticism, both the CSCL-11 and SCL-K-9 incorporate it.

The Cronbach’s alpha coefficients for these versions ranged from 0.87 to 0.94 in the total sample of this study, surpassing that for the CSCL-11 (α = 0.84). Table 7 summarizes the performance of various abbreviated versions in predicting the original GSI scores using the linear regression prediction model in the test set. The RMSEs for GSI predictions ranged from 0.08 to 0.13. R^2^ varied between 0.89 and 0.96, while Pearson’s r coefficients between predicted and actual values were between 0.94 and 0.98. Notably, when compared to other abbreviated versions, the CSCL-11 outperforms the SCL-14 (RMSE = 0.12), SCL-K11 (RMSE = 0.13), and SCL-K-9 (RMSE = 0.13) by demonstrating a lower RMSE of 0.11, which indicates that the model has a high level of accuracy when predicting the original GSI scores. The CSCL-11 has a robust R^2^ of 0.92 compared to the SCL-14 (R^2^ = 0.90), SCL-K11 (R^2^ = 0.89) and SCL-K-9 (R^2^ = 0.89), which demonstrates its ability to explain the variance in the data and outperforms the SCL-14, SCL-K11 and SCL-K-9. A Pearson’s r of 0.96 indicates a strong positive correlation with the predicted versus actual scores for CSCL-11, also outperforming the SCL-14, SCL-K11 and SCL-K-9.

**Figure 4:**
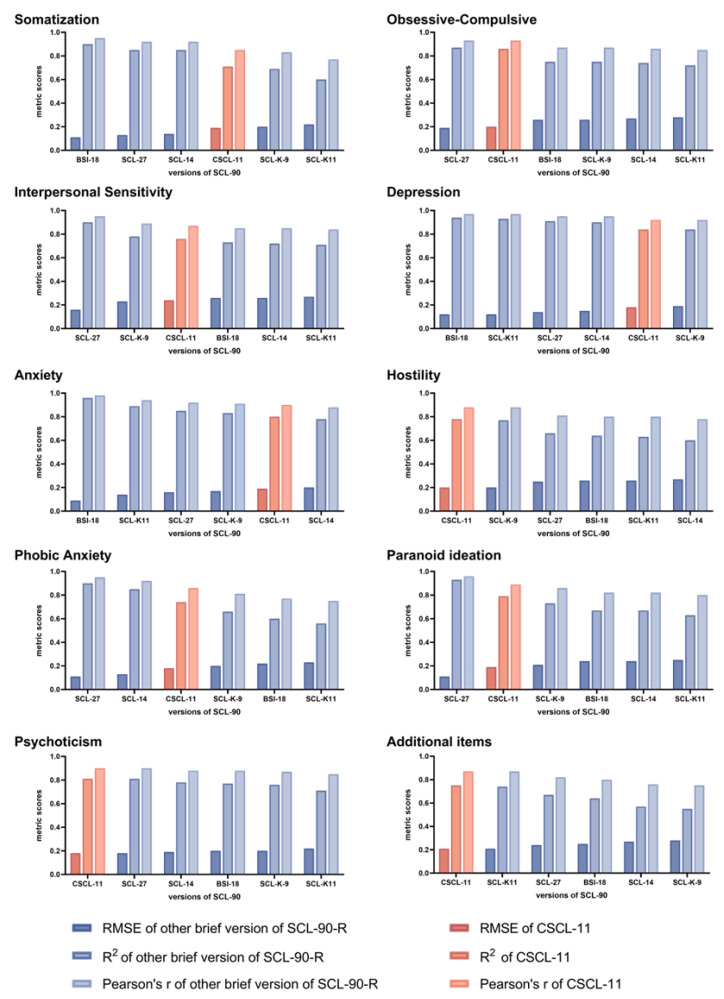
Visualization of the predictive scores of all mentioned shortened versions for dimensional assessments.

Additionally, Table 8 compares the performance of each version of the SCL-90-R in predicting subscale scores with visualizations presented in Figure 4. Except for the SCL-K-9 and CSCL-11, which have consistent performance in all the dimensions, other simplified versions exhibited a tendency to have varying predictive performance across the dimensions. In the hostility dimension and additional items, the CSCL-11’s predictive performance surpassed all other abbreviated versions (hostility: RMSE = 0.20, R^2^ = 0.78, r = 0.88; additional items: RMSE = 0.21, R^2^ = 0.75, r = 0.87). For psychoticism, its performance was comparable to that of SCL-27 (RMSE = 0.18, R^2^ = 0.81, r = 0.90) and superior to other versions. It also performed well in the obsessive-compulsive and paranoid ideation dimensions (obsessive-compulsive: RMSE = 0.20, R^2^ = 0.86, r = 0.93; paranoid ideation: RMSE = 0.19, R^2^ = 0.79, r = 0.89), surpassing all other brief versions except for SCL-27 (obsessive-compulsive: RMSE = 0.19, R^2^ = 0.87, r = 0.93; paranoid ideation: RMSE = 0.11, R^2^ = 0.93, r = 0.96). The CSCL-11 maintained average predictive performance in somatization, interpersonal sensitivity, and phobic anxiety dimensions (somatization: RMSE = 0.19, R^2^ = 0.71, r = 0.85; interpersonal sensitivity: RMSE = 0.24, R^2^ = 0.76, r = 0.87; phobic anxiety: RMSE = 0.18, R^2^ = 0.74, r = 0.86). However, in the depression and anxiety dimensions, the predictive performance of the CSCL-11 was slightly inferior to most of the other abbreviated versions but still satisfactory. Its prediction results for depression (RMSE = 0.18, R^2^ = 0.84, r = 0.92) only exceeded SCL-K-9 (RMSE = 0.19, R^2^ = 0.84, r = 0.92). For the anxiety dimension, its predictive performance (RMSE = 0.19, R^2^ = 0.80, r = 0.90) only surpassed SCL-14 (RMSE = 0.20, R^2^ = 0.78, r = 0.88).

To validate the feasibility of CSCL-11 as a preliminary psychopathological screening tool, we compared the performance of different brief versions in identifying the high-risk group for psychological disorders. Table 9 summarizes their accuracies, F1 scores, and Matthews correlation coefficients. The performance of CSCL-11 (accuracy = 0.96, F1 score = 0.81, MCC = 0.79) was similar to that of SCL-14, and outperformed both SCL-K11 (accuracy = 0.95, F1 score = 0.78, MCC = 0.75) and SCL-K-9 (accuracy = 0.95, F1 score = 0.79, MCC = 0.76).

Taken together, these predictive metrics underscore the practical significance of even modest improvements in model performance. In psychological screening, metrics such as RMSE, R^2^, and Pearson’s r serve as critical indicators of the predictive accuracy for GSI and dimension scores. Even minor improvements—such as a modest reduction in RMSE, slight increases in R^2^ and correlation coefficients, and overall enhanced prediction accuracy—reflect a higher degree of precision and reliability in assessing an individual’s mental health status. This is especially crucial in the early detection of conditions like depression, anxiety, and phobic disorders, where even small prediction errors can lead to misdiagnoses, delay timely interventions, and complicate subsequent treatment. Thus, while the observed differences among these metrics may appear minimal, they signify a substantial gain in the precision and trustworthiness of the screening predictions, which is essential for effective risk stratification and the development of targeted interventions.

### 3.4. Summary

The outcome of the statistical analysis is that CSCL-11 shows consistently strong performance across several dimensions and, therefore, is very likely to be useful for screening psychological distress. The scale has excellent predictive accuracy, as evidenced by all dimensions having low RMSE values. Additionally, CSCL-11 has high R^2^ values, especially for GSI (0.92), suggesting that CSCL-11 explains a lot of psychological distress, confirming its robustness. Pearson’s r values illustrate the strong correlation between predicted and actual scores, such as the r = 0.96 for GSI, which proves the scale reflects psychological distress. The reliability and precision (accuracy = 0.96) of the CSCL-11 make it an effective tool for detecting individuals at high risk for psychological disorders so that timely and accurate interventions can be provided. The CSCL-11 has excellent predictive accuracy and reliability, so there are no doubts that it could be implemented in mental health screening widely, providing an efficient solution to early detection and intervention in different populations.

## 4. Discussion

In this study, we utilized variable clustering to develop the CSCL-11, a preliminary screening tool specifically designed for large-scale psychological surveys in China. The results of EFA and CFA indicated that the CSCL-11 possesses a unidimensional structure. Additionally, the CSCL-11 exhibited substantial internal consistency (McDonald’s omega = 0.839, Cronbach’s α = 0.84). By utilizing the linear regression algorithm to predict original GSI scores and subscale scores, it was found that the CSCL-11 could sufficiently capture the information transmitted by the original SCL-90 and its subscales. Furthermore, the construction of a gradient boosting decision tree (GBDT) classifier model enabled the CSCL-11 to identify individuals at high risk for psychological disorders accurately.

Although the sample includes a higher proportion of female students, theoretical perspectives suggest that mental health outcomes—such as anxiety, depression, and stress—share similar underlying mechanisms across genders, implying that this imbalance is unlikely to substantially affect the overall conclusions. In comparison with other brief versions of SCL-90-R, the CSCL-11 achieves good performance in predicting original GSI scores and subscale scores, as well as identifying individuals at high risk of psychological disorders while maintaining a relatively small number of items. In the context of psychological screening, models with higher predictive accuracy can enhance the early identification of at-risk individuals and support more efficient allocation of mental health resources. For instance, CSCL-11’s robust predictive performance across multiple dimensions—such as hostility and psychoticism—suggests its potential utility as a reliable and efficient tool for large-scale mental health assessments. The findings of this study suggested that variable clustering might be a good simplification algorithm for lengthy scales. Given that the structure of SCL-90 was previously found to be inconsistent, the use of variable clustering in this study can help break the dimensional limitations of the SCL-90 and focus more directly on the correlations among items. This approach is superior to the Principal Component Analysis (PCA) algorithm used in other SCL-90 simplifications (e.g., SCL-27, BSI-18). PCA reduces dimensionality by creating composite components that maximize explained variance but do not take into account the correlated structure among items. This approach can potentially lead to the loss of information related to the underlying correlation structure (Muñoz-Pichardo et al., 2024). In contrast, variable clustering constructs a correlation matrix to cluster items with similar characteristics together, ensuring that the representative item selected reflects the original correlated structure of the cluster. Moreover, the PCA only considers the relationship between selected items and the items within their own principal components. In contrast, variable clustering uses the RS_Ratio to select representative items, ensuring that the chosen items are highly correlated with items within their own cluster while maintaining minimal correlation with items from other clusters. At the same time, the chosen items in variable clustering were kept as dissimilar from each other as possible, resulting in a slightly lower internal consistency of the CSCL-11 (α = 0.84) compared to other simplified versions (α = 0.87~0.94). However, compared to the SCL-27, BSI-18, SCL-14, and SCL-K11, the CSCL-11 achieves a more balanced prediction across various dimensions without significant biases. Although the SCL-K-9 also demonstrates good predictive effectiveness across nine primary dimensions, our approach differs as it simplifies the scale from a holistic perspective, breaking dimensional constraints and preserving the information conveyed by correlations from all items in the original whole scale. Although the CSCL-11 did not include items from the interpersonal sensitivity subscale, its subscale scores can be well predicted from the existing items, which further demonstrated the feasibility of variable clustering in SCL-90 simplification. Furthermore, the excellent performance of CSCL-11 as a screening tool for identifying individuals at high risk for psychological disorders suggested the superiority of applying a machine learning classifier in linking the selected subset of items to the high-risk group identification obtained from the 90 items in the original SCL-90. In this study, the GBDT algorithm was considered the optimal classifier due to its excellent performance in handling nonlinear and highly correlated data. The GBDT’s iterative approach enables adaptive refinement of predictions by correcting previous errors, thereby enhancing its ability to model complex relationships between variables. Additionally, its robustness to noise and outliers ensures reliable and consistent results, making it particularly well-suited for our analysis. The GBDT algorithm developed in this study is also provided along with the CSCL-11 for future rapid identification of high-risk groups.

The 11-item CSCL-11 serves as a cost-effective and efficient screening tool by significantly reducing the number of items from the original Chinese version of SCL-90 without sacrificing predictive validity or reliability. This shortened scale is particularly advantageous for large-scale screenings where resources and time are limited. It demonstrates strong predictive performance, especially in identifying individuals at high risk of developing mental disorders, thereby aiding in their proper allocation in screening settings and enhancing early detection.

## 5. Conclusions

This study successfully simplified the Chinese version of SCL-90 by creating the Chinese SCL-11 (CSCL-11). The findings indicated that the CSCL-11 exhibited high internal consistency as a unidimensional scale and effectively captured the majority of the information from the original Chinese SCL-90. Additionally, it achieved a 96% accuracy rate in identifying individuals at high risk for psychological disorders. Following the comparative validation against previous brief versions of SCL-90-R, CSCL-11 exhibited performance similar to that of SCL-14 and additionally surpassed both SCL-K-9 and SCL-K11.

Despite the excellent predictive performance of CSCL-11, there were several limitations to this study. First, the research sample was drawn exclusively from students of a single institution, resulting in low sample variance that may affect the generalizability of the findings to broader populations and limit the applicability of the results. Moreover, the gender imbalance inherent in the institution—where the male-to-female ratio is uneven—may further compromise the scale’s universal applicability. Future research should aim to validate the effectiveness and stability of the CSCL-11 across different populations, including those with more balanced gender distributions. Additionally, potential biases in the data collection process, such as the subjectivity of self-reported responses and the influence of participants’ psychological states, could affect the reliability of the results. To mitigate these biases, future studies could consider using multiple data sources for cross-validation, conducting longitudinal studies to test the predictive power of the CSCL-11 over time, and investigating its effectiveness in different cultural contexts. Another limitation of this study is that only variable clustering was used for item selection. Future research could explore other feature selection methods, such as Recursive Feature Elimination (RFE) and the Boruta algorithm, to investigate whether different algorithms might yield different results. Although the GSI T-score ≥ 63 threshold has been widely applied in multiple studies, future research could compare different thresholds (e.g., T-score ≥ 60, ≥65) and explore alternative classification standards to determine the most suitable criterion for Chinese university students, thereby improving the accuracy and generalizability of mental health assessments.

## Figures and Tables

**Table 1:** Description of methods for predicting binary results.

Classifier Name	Description
LR	Logistic regression is a widely used supervised learning algorithm for binary classification. It employs the Sigmoid function to map the linear combination of features to a probability value between 0 and 1. The category is then predicted by a threshold, typically 0.5.
GBDT	This is an ensemble learning method comprised of multiple decision trees. The algorithm iteratively learns from residuals. It constructs new trees using the derivative of the loss function as a gradient, guiding tree growth to reduce residuals and diminish prediction errors.
ADA	ADA is an adaptive ensemble learning method that combines multiple ‘weak’ classifiers to construct an enhanced model. The algorithm iteratively adjusts the weights of samples during the training process to reduce the probability of misclassification.
RF	Random forest builds a Bagging ensemble with decision trees as the base learners and introduces randomness during the training process of the trees. The final output is determined by a voting method involving each tree in the forest.
SVC	SVC employs kernel functions to project datasets into a higher-dimensional space, where it identifies a hyperplane that maximizes the decision boundary between different categories. The algorithm assigns the class label of a new data point based on its proximity to this decision boundary.

Abbreviations: LR, logistic regression; GBDT, gradient boosting decision tree; ADA, adaptive boosting; RF, random forest; SVC, support vector classifier.

**Table 2:** The shortened version and its relationship with the items of the original SCL-90.

Number in SCL-90	Items	Dimension in SCL-90
8	Feeling others are to blame for most of your troubles	PAR
9	Trouble remembering things	OC
16	Hearing voices that other people do not hear	PSY
30	Feeling blue	DEP
33	Feeling fearful	ANX
45	Having to check and double-check what you do	OC
52	Numbness or tingling in parts of your body	SOM
63	Having urges to beat, injure, or harm someone	HOS
66	Sleep that is restless or disturbed	ADD
70	Feeling uneasy in crowds, such as shopping or at a movie	PHOB
85	The idea that you should be punished for your sins	PSY

**Table 3:** Factor loadings for EFA.

Number in SCL-90	Items	Factor Loading
8	Feeling others are to blame for most of your troubles	0.584
9	Trouble remembering things	0.575
16	Hearing voices that other people do not hear	0.425
30	Feeling blue	0.748
33	Feeling fearful	0.690
45	Having to check and double-check what you do	0.574
52	Numbness or tingling in parts of your body	0.541
63	Having urges to beat, injure, or harm someone	0.606
66	Sleep that is restless or disturbed	0.491
70	Feeling uneasy in crowds, such as shopping or at a movie	0.555
85	The idea that you should be punished for your sins	0.547

**Table 4:** The performance of the shortened version in predicting total scores and factor scores.

Dimensions	RMSE	R^2^	Pearson’s r (r)
GSI	0.11	0.92	0.96
SOM	0.19	0.71	0.85
OC	0.20	0.86	0.93
IS	0.24	0.76	0.87
DEP	0.18	0.84	0.92
ANX	0.19	0.80	0.90
HOS	0.20	0.78	0.88
PHOB	0.18	0.74	0.86
PAR	0.19	0.79	0.89
PSY	0.18	0.81	0.90
ADD	0.21	0.75	0.87

**Table 5:** Results of model evaluation for identifying the high-risk group. Results are shown as average values with corresponding 95% CI.

Models	Accuracy	Sensitivity/Recall	Specificity	Precision	F1 Score	MCC
Logistic regression (LR)	0.96 [0.95, 0.97]	0.76 [0.70, 0.81]	0.98 [0.98, 0.99]	0.85 [0.79, 0.90]	0.80 [0.76, 0.83]	0.78 [0.74, 0.82]
Random forest (RF)	0.96 [0.95, 0.96]	0.70 [0.64, 0.75]	0.99 [0.98, 0.99]	0.85 [0.80, 0.90]	0.76 [0.71, 0.81]	0.75 [0.69, 0.80]
Adaptive boosting (Adaboost)	0.96 [0.96, 0.97]	0.79 [0.75, 0.83]	0.98 [0.98, 0.99]	0.84 [0.80, 0.89]	0.81 [0.78, 0.85]	0.79 [0.76, 0.83]
Gradient boosting decision tree (GBDT)	0.96 [0.95, 0.97]	0.78 [0.73, 0.83]	0.98 [0.98, 0.99]	0.84 [0.80, 0.88]	0.81 [0.77, 0.84]	0.79 [0.75, 0.83]
Support vector classifier (SVC)	0.93 [0.92, 0.94]	0.57 [0.52, 0.63]	0.98 [0.97, 0.98]	0.76 [0.70, 0.82]	0.65 [0.60, 0.70]	0.62 [0.57, 0.67]

**Table 6:** Dimensional structure of the Chinese version of SCL-11, SCL-27, BSI-18, SCL-14, and SCL-K-9. (The checkmarks (✓) indicate that the abbreviated scale includes items from the corresponding subscale).

SCL-90 Subscales	CSCL-11	SCL-27	BSI-18	SCL-14	SCL-K11	SCL-K-9
SOM	✓	✓	✓	✓		✓
OC	✓	✓				✓
IS		✓				✓
DEP	✓	✓	✓	✓	✓	✓
ANX	✓	✓	✓		✓	✓
HOS	✓					✓
PHOB	✓	✓		✓		✓
PAR	✓	✓				✓
PSY	✓					✓
ADD	✓	✓			✓	

**Table 7:** Predictive scores (RMSE, R^2^, and Pearson’s r) of all mentioned shortened versions for GSI scores.

Versions of SCL-90	Cronbach’s Alpha	RMSE	R^2^	Pearson’s r (r)
CSCL-11	0.84	0.11	0.92	0.96
SCL-27	0.94	0.08	0.96	0.98
BSI-18	0.92	0.10	0.93	0.97
SCL-14	0.90	0.12	0.90	0.95
SCL-K11	0.90	0.13	0.89	0.94
SCL-K-9	0.87	0.13	0.89	0.95

**Table 8:** Predictive scores (RMSE, R^2^, and r) of all mentioned shortened versions for dimensional assessments.

SCL-90 Subscales	CSCL-11			SCL-27			BSI-18			SCL-14			SCL-K11			SCL-K-9		
Metrics	RMSE	R^2^	r	RMSE	R^2^	r	RMSE	R^2^	r	RMSE	R^2^	r	RMSE	R^2^	r	RMSE	R^2^	r
SOM	0.19	0.71	0.85	0.13	0.85	0.92	**0.11**	**0.90**	**0.95**	0.14	0.85	0.92	0.22	0.60	0.77	0.20	0.69	0.83
OC	0.20	0.86	0.93	**0.19**	**0.87**	**0.93**	0.26	0.75	0.87	0.27	0.74	0.86	0.28	0.72	0.85	0.26	0.75	0.87
IS	0.24	0.76	0.87	**0.16**	**0.90**	**0.95**	0.26	0.73	0.85	0.26	0.72	0.85	0.27	0.71	0.84	0.23	0.78	0.89
DEP	0.18	0.84	0.92	0.14	0.91	0.95	**0.12**	**0.94**	**0.97**	0.15	0.90	0.95	0.12	0.93	0.97	0.19	0.84	0.92
ANX	0.19	0.80	0.90	0.16	0.85	0.92	**0.09**	**0.96**	**0.98**	0.20	0.78	0.88	0.14	0.89	0.94	0.17	0.83	0.91
HOS	**0.20**	**0.78**	**0.88**	0.25	0.66	0.81	0.26	0.64	0.80	0.27	0.60	0.78	0.26	0.63	0.80	0.20	0.77	0.88
PHOB	0.18	0.74	0.86	**0.11**	**0.90**	**0.95**	0.22	0.60	0.77	0.13	0.85	0.92	0.23	0.56	0.75	0.20	0.66	0.81
PAR	0.19	0.79	0.89	**0.11**	**0.93**	**0.96**	0.24	0.67	0.82	0.24	0.67	0.82	0.25	0.63	0.80	0.21	0.73	0.86
PSY	**0.18**	**0.81**	**0.90**	**0.18**	**0.81**	**0.90**	0.20	0.77	0.88	0.19	0.78	0.88	0.22	0.71	0.85	0.20	0.76	0.87
ADD	**0.21**	**0.75**	**0.87**	0.24	0.67	0.82	0.25	0.64	0.80	0.27	0.57	0.76	0.21	0.74	0.87	0.28	0.55	0.75

The bold text indicates the abbreviated version that performed best in that dimension.

**Table 9:** Predictive performance (accuracy, F1 score, and MCC) of all mentioned shortened versions for identifying the high-risk group for psychological disorders.

Versions of SCL-90	Accuracy	F1 Score	Matthews Correlation Coefficient
CSCL-11	0.96	0.81	0.79
SCL-27	0.97	0.87	0.86
BSI-18	0.97	0.84	0.83
SCL-14	0.96	0.81	0.79
SCL-K11	0.95	0.78	0.75
SCL-K-9	0.95	0.79	0.76

## Data Availability

The study data and the developed machine learning classifier were available from the corresponding author upon request.

## Abbreviations

The following abbreviations are used in this manuscript:

SCL-90	Symptom Checklist 90
SCL-90-R	Symptom Checklist-Revised-90
BSI	Brief Symptom Inventory
BSI-18	Brief Symptom Inventory-18
CSCL-11	Chinese SCL-11
GSI	Global Severity Index
SOM	Somatization
OC	Obsessive-Compulsive
IS	Interpersonal Sensitivity
DEP	Depression
ANX	Anxiety
HOS	Hostility
PHOB	Phobic Anxiety
PAR	Paranoid Ideation
PSY	Psychoticism
ADD	Additional items
RMSE	Root Mean Square Error
R2	Coefficient of Determination
Pearson’s r	Pearson Correlation Coefficient
α	Cronbach’s Alpha Coefficient
EFA	Exploratory Factor Analysis
CFA	Confirmatory Factor Analysis
SRMR	Standardized Root Mean Squared Residual
RMSEA	Root Mean Squared Error of Approximation
CFI	Comparative Fit Index
TLI	Tucker–Lewis Index
MCC	Matthews Correlation Coefficient
ROC	Receiver Operating Characteristic
PRC	Precision–Recall Curves
LR	Logistic Regression
GBDT	Gradient Boosting Decision Tree
GBRT	Gradient Boosting Regression Tree
ADA	Adaptive Boosting
RF	Random Forest
SVC	Support Vector Classifier
